# Diesel exhaust particles induce human umbilical vein endothelial cells apoptosis by accumulation of autophagosomes and caspase-8 activation

**DOI:** 10.1038/s41598-022-21044-3

**Published:** 2022-10-03

**Authors:** Geun-Young Kim, Inkyo Jung, Minhan Park, Kihong Park, Seung Hee Lee, Won-Ho Kim

**Affiliations:** 1 Division of Cardiovascular Disease Research, Department of Chronic Disease Convergence Research, Korea National Institute of Health, 187 Osongsaengmyeng2-ro, Osong-eub, Heungdeok-gu, Cheongju-si, Chungcheongbuk-do 28159 Republic of Korea; 2 School of Earth Science and Environmental Engineering, Gwangju Institute of Science and Technology, Gwangju, Republic of Korea

**Keywords:** Cell biology, Molecular biology

## Abstract

Diesel exhaust particles (DEP) are risk factors for endothelial cells (ECs) dysfunction. However, the mechanism by which DEP induce ECs apoptosis remains unclear. Here, we investigated how DEP induce death of human umbilical vein ECs (HUVECs), with a focus on the autophagy-mediated apoptotic pathway. DEP induced dose-dependent HUVECs death and exposure to the IC_50_ concentration of DEP (70 µg/ml) led to apoptosis. DEP phosphorylated Beclin-1 (Ser93) and increased protein levels of p62 and LC3BII and the number of LC3B puncta, indicating autophagy initiation. DEP increased expression of pro- and mature forms of cathepsin D, which increases lysosomal activity. However, DEP suppressed expression of the soluble N-ethylmaleimide-sensitive factor attachment protein receptor proteins (STX17, VAMP8, SNAP29, YKT6, and STX7) to inhibit autolysosome formation, resulting in accumulation of autophagosomes. LC3B, p62, and caspase-8 form a tertiary complex in accumulated autophagosomes, which is known to serve as a platform for caspase-8 activation. Indeed, DEP activates caspase-8 and pretreatment with a caspase-8 inhibitor suppressed DEP-induced apoptosis. Furthermore, depletion of p62 decreased caspase-8 and caspase-3 activation and inhibited the DEP-induced apoptosis. Taken together, these findings demonstrated that DEP induced HUVECs apoptosis by inhibiting autophagosome maturation and identified caspase-8 as a novel mediator of DEP-induced ECs apoptosis.

## Introduction

Autophagy is the process by which cytosolic materials, regardless of their origin, are sequestered into the autophagosome and delivered to the lysosome for degradation in response to environmental or physiological stressors^1,2^. For completion of the autophagic process, autophagosomes fuse with lysosomes in the process of autophagosome maturation^2^. Previous studies have identified regulatory proteins for this process, such as soluble N-ethylmaleimide-sensitive factor attachment protein receptor (SNARE) complexes and their post-translational or transcriptional modifications^2–6^. However, the machinery for autophagosome maturation depends on the autophagy-inducing stimuli and cell type^2^.

Particulate matter (PM), comprised of toxic chemicals and particles, increases the risk of multiple diseases and premature death worldwide^7,8^. PM2.5, which enters the body through the respiratory system and has a diameter < 2.5 μm, directly penetrates blood vessels and circulates in the body, adversely affecting almost all tissues^9^. Interestingly, although the respiratory system is the first organ exposed to PM, the adverse effects of PM2.5 are most often observed in cardiovascular disease^8^. Endothelial cells (ECs) line the interior surfaces of blood vessels and play a critical role in normal physiological events such as vascular tone by regulating endothelial-dependent smooth muscle relaxation^10^. Thus, ECs dysfunction is a risk factor for several vascular diseases, including hypertension and atherosclerosis^11,12^. Because PM causes ECs dysfunction by inducing inflammation or apoptosis^13,14^, investigations targeting the effects of PM on ECs are expected to yield insight into therapeutic approaches for PM-induced vascular diseases.

Caspase-8 is an initiator caspase, which is activated by recruitment to death receptors such as Fas/CD95 and TNF receptor-1 and associates with Fas-associated death domain (FADD) and TNFR1-associated death domain (TRADD) to form the death-inducing signaling complex (DISC)^15^. Further, caspase-8 can be activated in the autophagosomal membrane, which serves as a platform for intracellular DISC (iDISC) formation that enables caspase-8 activation^16^. PM induces apoptosis in various cell types^13,14,17^, but the involvement of caspase-8 in PM-induced apoptosis has not been investigated. Interestingly, diesel exhaust particles (DEP) induce ECs apoptosis by increasing p53 accumulation and inhibiting autophagy^18^. In addition, DEP induces NADPH-oxidase-dependent reactive oxygen species (ROS) generation, protein expression related to autophagy (p62, beclin-1 and LC3II), and apoptosis in HUVECs^19^. However, the precise mechanisms of these phenomena remain incompletely understood.

In the present study, we investigated the mechanism of DEP-induced apoptosis in human umbilical vein endothelial cells (HUVECs). We and others selected this cell type because many prior studies have demonstrated that HUVECs recapitulate phenomena that occur in blood vessels^13,18,20,21^. HUVECs exposure to DEP initiated the autophagic process, but blocked autophagosome maturation by suppressing expression of SNARE proteins. The accumulated autophagosomes induced p62/SQSTM1-dependent caspase-8 cleavage and cleaved caspase-8 induced downstream caspase-3 activation and subsequent apoptosis. Elucidating the mechanisms by which DEP modulate the autophagic process and regulate ECs apoptosis will contribute to development of new therapeutic interventions for PM-induced vascular disease.

## Results

### Chemical composition of DEP

DEP were subjected to analysis of mass fractions of chemical components (elements, ions, and carbonaceous species). DEP had the highest mass fraction of organic carbon (92.05%), followed by ions (2.12%), elemental carbon (0.52%), and elements (0.07%), including others (5.24%) (see Supplementary Table S1 online). P was the most abundant element (36.50%) in DEP, followed by Ca (15.96%) and Al (14.57%) (see Supplementary Table S2 online). NO_3_^-^ was the most abundant ion (60.65%), followed by Na^+^ (15.89%) and SO_4_^2−^ (14.86%) (see Supplementary Table S3 online).

### DEP induce HUVECs apoptosis

**Figure 1:**
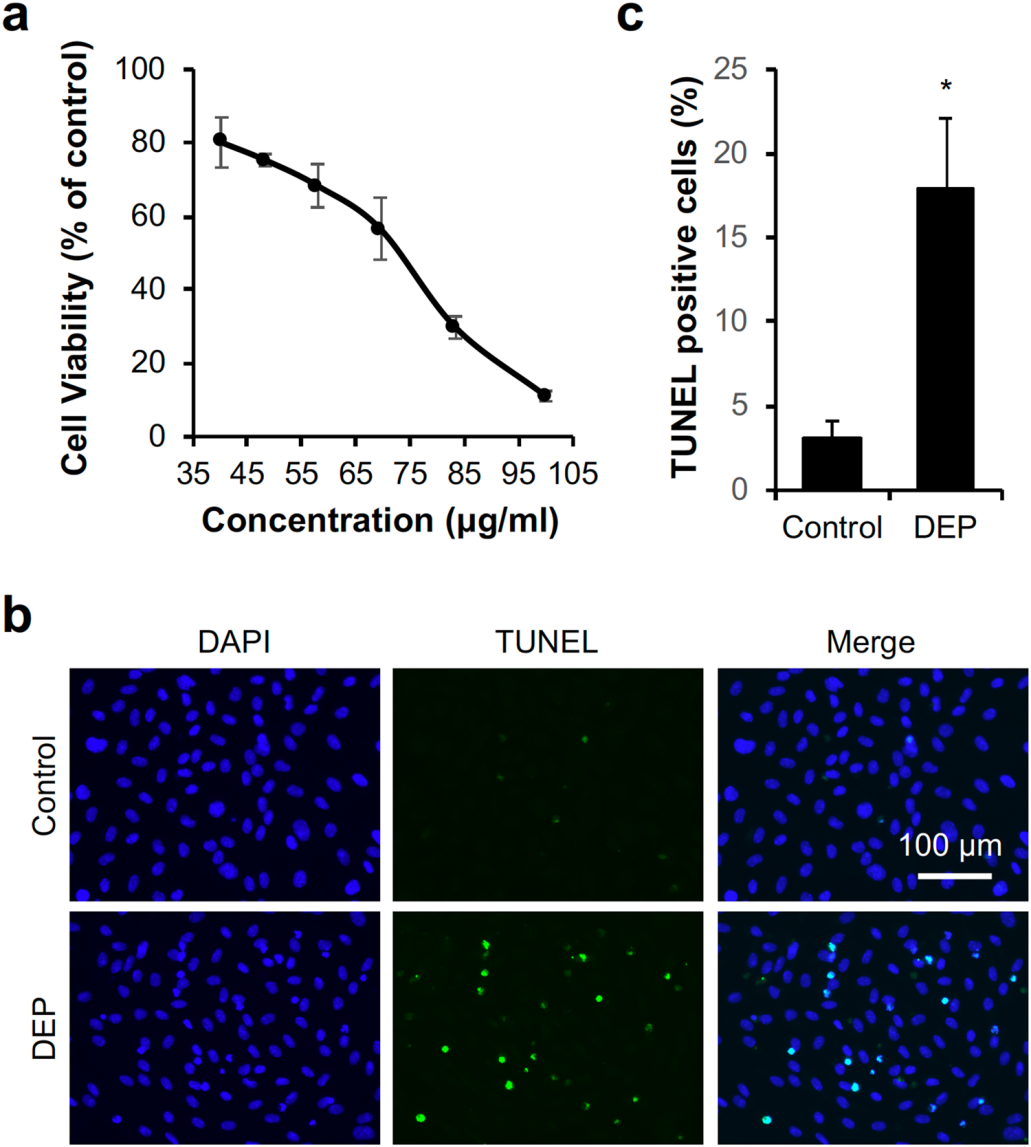
DEP induce HUVECs apoptosis. (**a**) HUVECs were exposed to various DEP concentrations for 24 h and cell viability was measured using a WST-1 assay. (**b**) HUVECs were exposed to the IC_50_ DEP concentration (70 µg/ml) for 24 h. Cells were then subjected to TUNEL staining. (**c**) Quantification of TUNEL-positive cells. TUNEL-positive apoptotic cells were counted and expressed as a percentage of total nuclear counts. Results are presented as means ± SD from five randomly selected fields. Statistical analysis was performed using two-tailed Student’s t test. *P < 0.05 versus control.

To determine the effect of DEP on cell viability, we treated HUVECs with various concentrations of DEP or DMSO (solute control) and measured cell viability after incubation for 24 h. Relative to DMSO-treated cells, DEP induced cell death in a dose-dependent manner (Fig. 1a) and the IC_50_ concentration (70 μg/ml) was subsequently used to investigate the relevant mechanisms of action. To determine whether the decrease in cell viability was due to apoptosis, we performed TUNEL analysis after exposing cells to DEP. DEP significantly increased the percentage of TUNEL-positive cells, indicating increased apoptosis (Fig. 1b and c).

### DEP initiate autophagy in HUVECs

**Figure 2:**
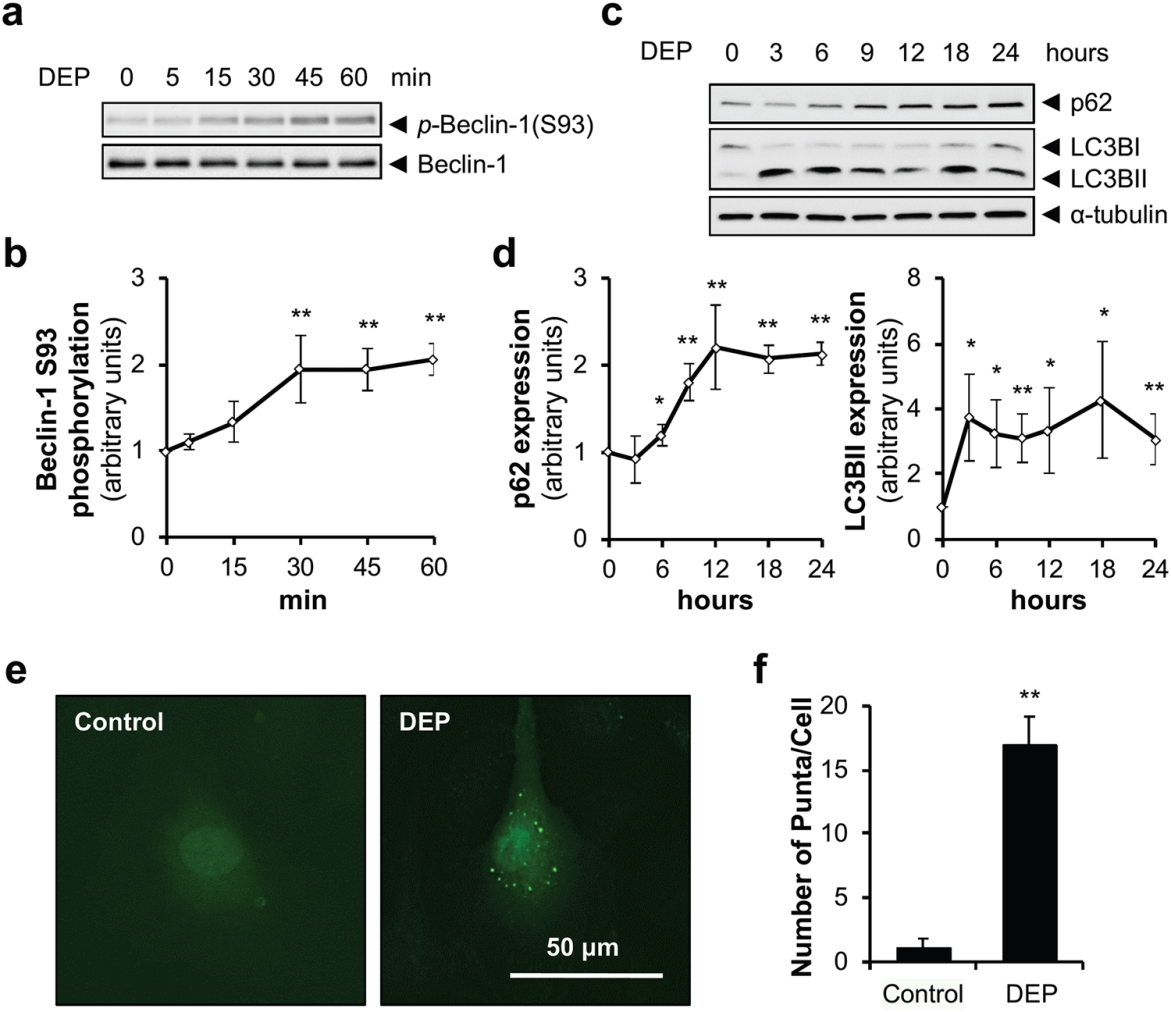
DEP initiate autophagy in HUVECs. (**a**) HUVECs were exposed to DEP (70 µg/ml) for the indicated times and phosphorylation of Beclin-1 at Ser93 was analyzed by immunoblotting. (**b**) Quantification of Beclin-1 phosphorylation normalized to total Beclin-1. Results are presented as means ± SD (n = 3). Statistical analysis was performed using one-way ANOVA. **P < 0.02 versus no treatment. (**c**) HUVECs were exposed to DEP (70 µg/ml) for the indicated times and protein levels of p62 and LC3B were measured by immunoblotting. (**d**) Quantification of p62 and LC3BII protein levels normalized to ⍺-tubulin. Results are presented as means ± SD (*n* = 3). Statistical analysis was performed using one-way ANOVA. *P < 0.05 versus no treatment. **P < 0.02 versus no treatment. (**e**) HUVECs infected with GFP-LC3B-expressing adenovirus were exposed to DEP (70 µg/ml) for 18 h and formation of LC3B puncta was examined with fluorescence microscopy. (**f**) Quantification of LC3B puncta formation per cell. Results are presented as means ± SD (*n* = 10). Statistical analysis was performed using two-tailed Student’s t test. **P < 0.02 versus control.

Previous studies have shown that autophagy can activate apoptotic pathways^22,23^, and that DEP with different sources than ours can induce autophagy^18,24^. Thus, we examined whether our DEP could initiate autophagic processes that are expected to induce apoptosis. Because phosphorylation of beclin-1 at serine 93 is required for autophagy^25,26^, we hypothesized that DEP would induce beclin-1 phosphorylation at serine 93. Consistent with our hypothesis, DEP induced beclin-1 phosphorylation (ser-93) (Fig. 2a and b), accompanied by progressive increases of p62 and LC3BII (Fig. 2c and d), known marker proteins for autophagy^27^. In addition, DEP exposure increased the number of GFP-LC3B puncta (Fig. 2e and f), a well-characterized marker of autophagosomes^27^. Taken together, these results indicated that DEP initiated the autophagic process and autophagosome formation in HUVECs.

### DEP increase lysosomal activity

**Figure 3:**
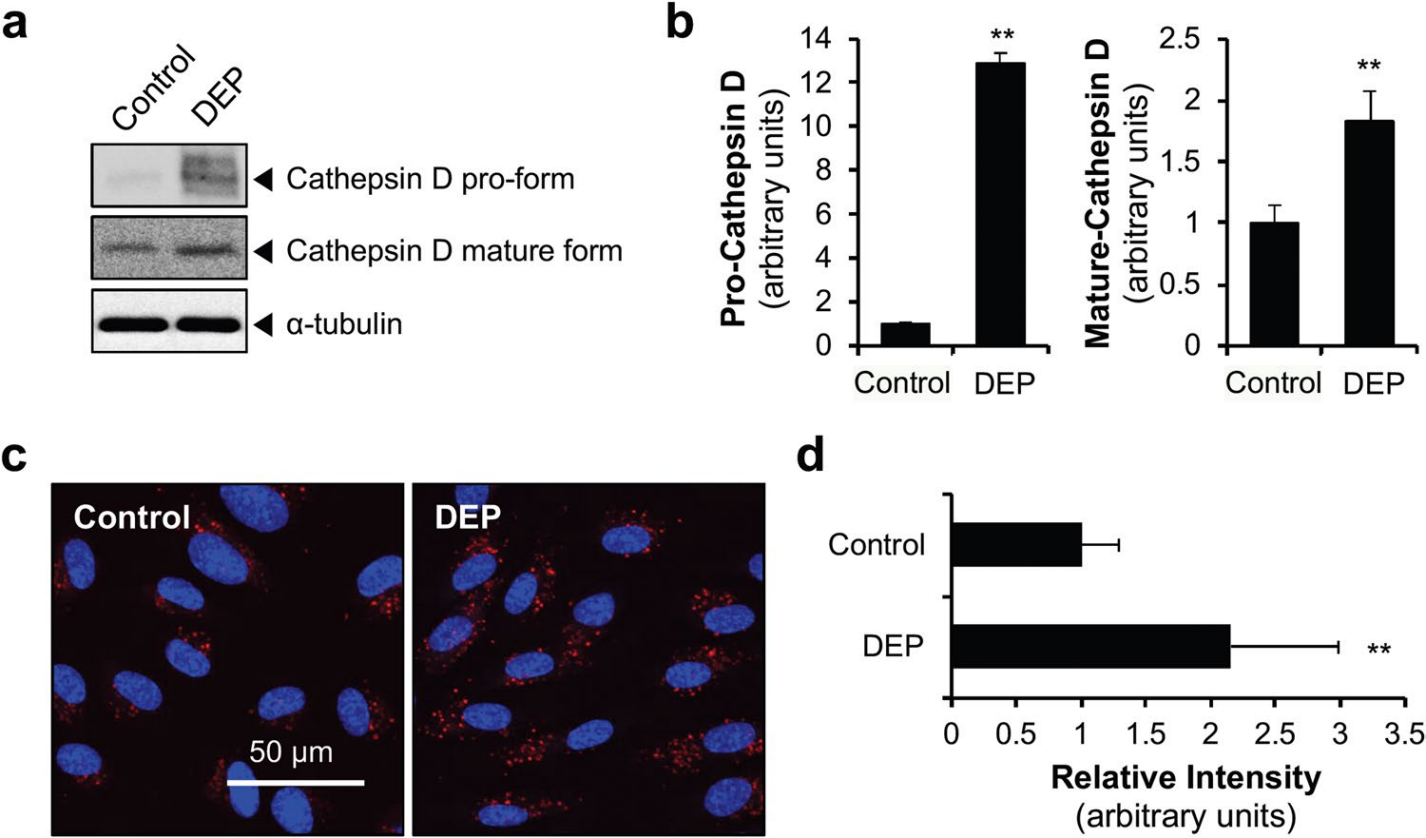
DEP increase lysosomal activity in HUVECs. (**a**) HUVECs were exposed to DEP (70 µg/ml) for 24 h and protein levels of cathepsin D (pro- and mature forms) were measured by immunoblotting. (**b**) Quantification of pro- and mature cathepsin D normalized to ⍺-tubulin. Results are presented as means ± SD (*n* = 3). Statistical analysis was performed using two-tailed Student’s t test. **P < 0.02 versus control. (**c**) After exposure to DEP for 12 h, cells were subjected to lysosome activity assay using DQ™ Red BSA. Lysosomal activity, which is reflected as red fluorescent products, was observed under confocal microscopy. (**d**) The relative intensity of red fluorescent products of each cell was quantified using Image J software (ver 1.53). Results are presented as means ± SD (n = 10). Statistical analysis was performed using two-tailed Student’s t test. **P < 0.02 versus control.

For completion of the autophagic process, the autophagosome must fuse with a lysosome to form an autolysosome, in which lysosomal enzymes degrade the cytoplasmic components^2^. As we observed DEP-induced autophagosome formation, we next examined whether DEP exposure affects lysosomal activity by regulating expression of the lysosomal enzyme cathepsin D. DEP exposure significantly increased the expression of both pro-cathepsin D and mature cathepsin D (Fig. 3a and b). We further analyzed lysosomal activity using DQ-BSA (Dye-Quenched Bovine Serum Albumin), in which degradation by hydrolases in active endo-lysosomes results in de-quenching of the dye and red fluorescence^28,29^. Consistent with the increased expression of lysosomal enzymes, DEP increased lysosomal activity (Fig. 3c and d). These findings suggest that in HUVECs exposed to DEP, lysosomal activity increases to initiate the autophagic process and complete autophagosome maturation.

### DEP impair autophagic flux and increase autophagosome accumulation

**Figure 4:**
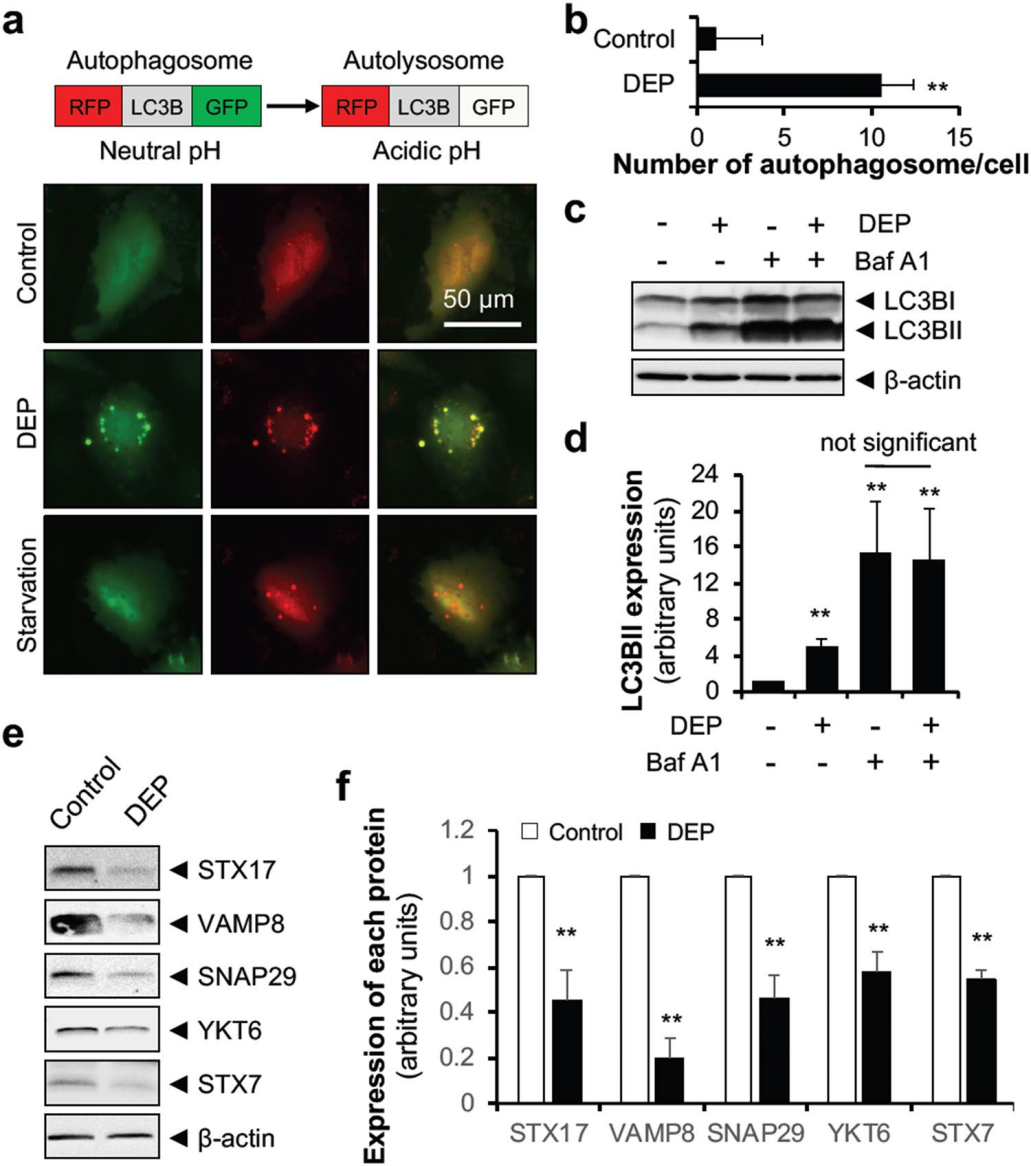
DEP impair autophagic flux in the degradation stage. (**a**) HUVECs infected with RFP-LC3B-GFP-expressing adenovirus were exposed to DEP (70 µg/ml) for 18 h and autolysosome formation was examined under fluorescence microscopy. (**b**) The number of yellow signals per cell was counted from 10 randomly selected cells. Statistical analysis was performed using two-tailed Student’s t test. **P < 0.02 versus control. (**c**) HUVECs were pre-incubated with Bafilomysin A1 (Baf A1, 100 nM) for 3 h and exposed to DEP (70 μg/ml) for an additional 12 h. LC3B level was measured by immunoblotting. (**d**) Quantification of LC3BII levels normalized to β-actin. Results are presented as means ± SD (*n* = 3). Statistical analysis was performed using one-way ANOVA. **P < 0.02 versus no treatment. (**e**) HUVECs were exposed to DEP (70 μg/ml) for 24 h and protein levels of STX17, VAMP8, SNAP29, YKT6, STX7, and β-actin were measured by immunoblotting. (**f**) Quantification of STX17, VAMP8, SNAP29, YKT6, and STX7 levels normalized to β-actin. Results are presented as means ± SD (*n* = 3). Statistical analysis was performed using two-tailed Student’s t test. **P < 0.02 versus control.

After observing autophagosome formation (Fig. 2e and f) and lysosomal activation (Fig. 3), we examined whether autophagosomes and lysosomes fused to form an autolysosome. To do this, we transduced HUVECs with adenovirus expressing RFP-LC3B-GFP and exposed transduced cells to DEP. GFP function is impaired in acidic conditions, so green fluorescence diminishes when autophagosomes fuse with the lysosomes, leaving only red fluorescence (RFP), which is indicative of autolysosomes (acidic pH)^30^. However, autophagosomes (neutral pH) can be visualized by both green and red fluorescence and when merged, produce a yellow signal^30^. When HUVECs were starved, only red fluorescence was present, indicating autolysosome formation (Fig. 4a). However, DEP exposure increased both green fluorescence and red fluorescence, indicating that autophagy was blocked at the autophagosome stage (Fig. 4a and b). Furthermore, an LC3 turnover assay demonstrated that when compared with cells treated with Bafilomycin A1 alone, there was no further increase in LC3BII level in both DEP and Bafilomycin A1 co-treated groups (Fig. 4c and d). This result of LC3 turnover assay was also observed in HAECs (Human Aortic Endothelial Cells) (see Supplementary Fig. S1 online), suggesting that although DEP initiate autophagy and increase lysosome activity, the autophagic process is blocked in the autophagosome stage, impairing completion of autophagic flux. Fusion between autophagosomes and lysosomes is mediated by two sets of SNARE proteins, the syntaxin 17 (STX17)–synaptosomal-associated protein 29 (SNAP29)–vesicle-associated membrane protein 8 (VAMP8) complex and the YKT6–SNAP29–STX7 complex^2–4,31^. Thus, we examined whether DEP affected levels of these proteins. DEP exposure significantly inhibited expression of STX17, VAMP8, SNAP29, YKT6, and STX7 in HUVECs (Fig. 4e and f) and HAECs (see Supplementary Fig. S2 online), suggesting that a possible mechanism of DEP-induced autophagosome accumulation is the inhibition of SNARE proteins expression.

### Caspase-8 activation is critical for DEP-induced apoptosis

**Figure 5:**
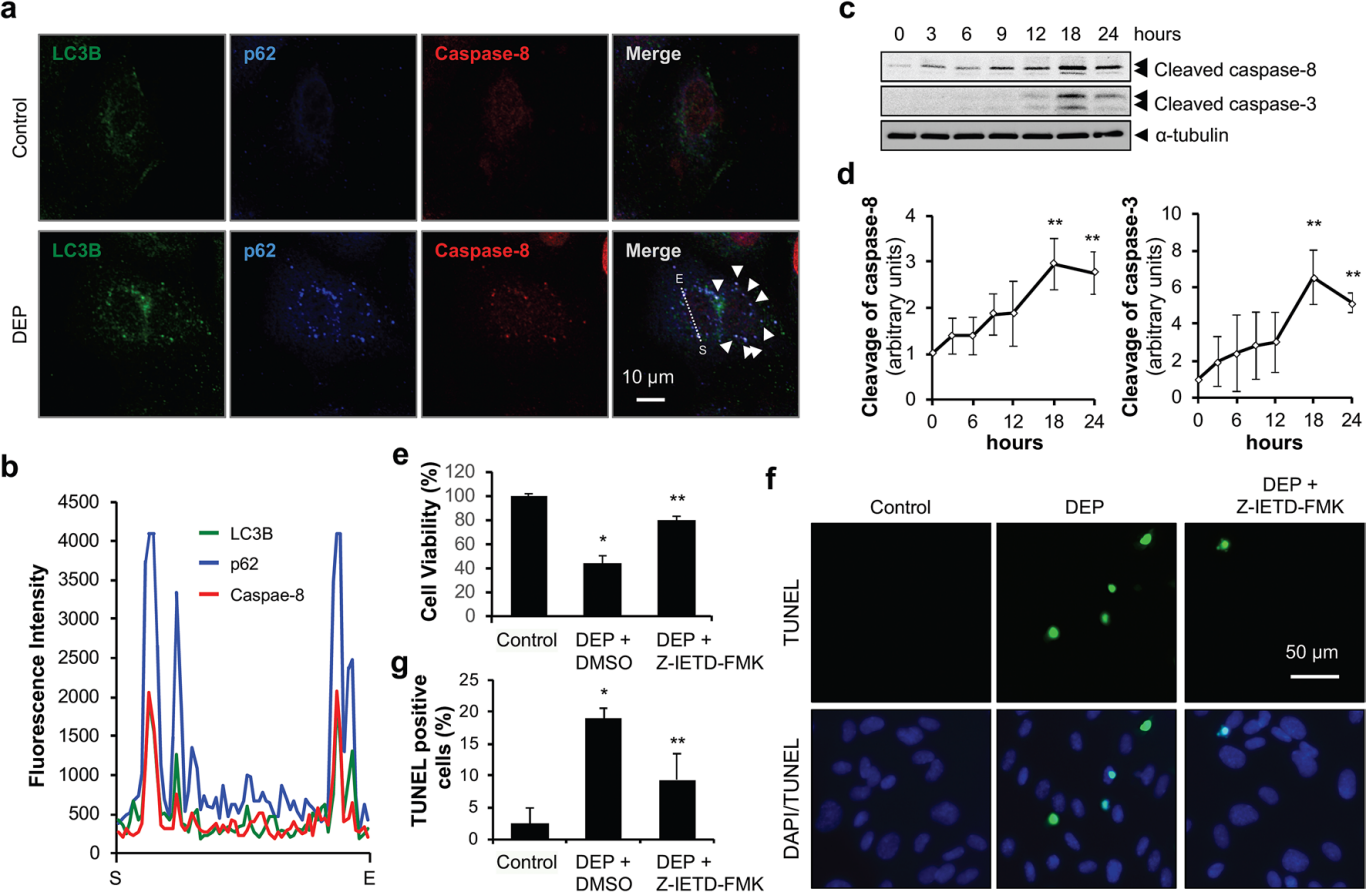
Caspase-8 is critical for DEP-induced apoptosis in HUVECs. (**a**) HUVECs were exposed to DEP (70 μg/ml) for 6 h. Cells were then fixed, permeabilized, and stained for LC3B, p62, and caspase-8. Arrows indicate co-localization between LC3B, p62, and caspase-8. (**b**) Intensity of each fluorescent signal along the dotted line shown in (**a**). S: Start, E: End. (**c**) HUVECs were exposed to DEP (70 µg/ml) for the indicated times and protein levels of cleaved caspase-8 and cleaved caspase-3 were analyzed by immunoblotting. (**d**) Quantification of cleaved caspase-8 and cleaved caspase-3 protein levels normalized to ⍺-tubulin. Results are presented as means ± SD (*n* = 3). Statistical analysis was performed using one-way ANOVA. **P < 0.02 versus no treatment. (**e**) HUVECs were pre-incubated with either Z-IETD-FMK (caspase-8 inhibitor, 5 µM) or vehicle control (DMSO) for 30 min and exposed to DEP (70 µg/ml) for 24 h. After performing WST-1 assay, cell viability relative to control is presented. Results are presented as means ± SD (*n* = 8). Statistical analysis was performed using one-way ANOVA. *P < 0.02 versus control. **P < 0.02 versus DEP + DMSO. (**f**) HUVECs were pre-incubated either with Z-IETD-FMK (5 µM) or vehicle control (DMSO) for 30 min and exposed to DEP (70 µg/ml) for 24 h. Cells were then subjected to TUNEL staining. (**g**) Quantification of TUNEL-positive cells. TUNEL-positive apoptotic cells were counted and expressed as percentage of total nuclear counts. Results are presented as means ± SD from five randomly selected fields. Statistical analysis was performed using one-way ANOVA. *P < 0.02 versus control. **P < 0.02 versus DEP + DMSO.

We next sought to determine the mechanism by which autophagosome accumulation induced apoptosis. Previous studies have identified the autophagic machinery required for cell death^22,32^, demonstrating that p62 bound to LC3 translocates caspase-8 to the autophagosome and induces activation^16,33^. Under these conditions, p62, LC3, and caspase-8 were observed to interact in autophagosomes to induce apoptosis^16,33^. Because we observed that DEP-induced cell death was apoptotic (Fig. 1), we examined the interaction between p62, LC3B, and caspase-8. DEP exposure induced assembly of a tertiary complex between p62, LC3B, and caspase-8 (Fig. 5a and b), suggesting that caspase-8 could be involved in DEP-induced apoptosis. We thus exposed HUVECs to DEP over a time course and examined activation (cleavage) of caspase-8 and its downstream target caspase-3. As expected, DEP exposure activated caspase-8 and caspase-3 (Fig. 5c and d). Furthermore, when HUVECs were pretreated with the caspase-8 inhibitor Z-IETD-FMK prior to DEP exposure, cell viability increased (Fig. 5e) and DEP-induced apoptosis decreased (Fig. 5f and g). These findings demonstrated that DEP exposure activates the caspase cascade associated with caspase-8–caspase-3 and leads to HUVECs apoptosis.

### p62 depletion suppresses DEP-induced apoptosis

**Figure 6:**
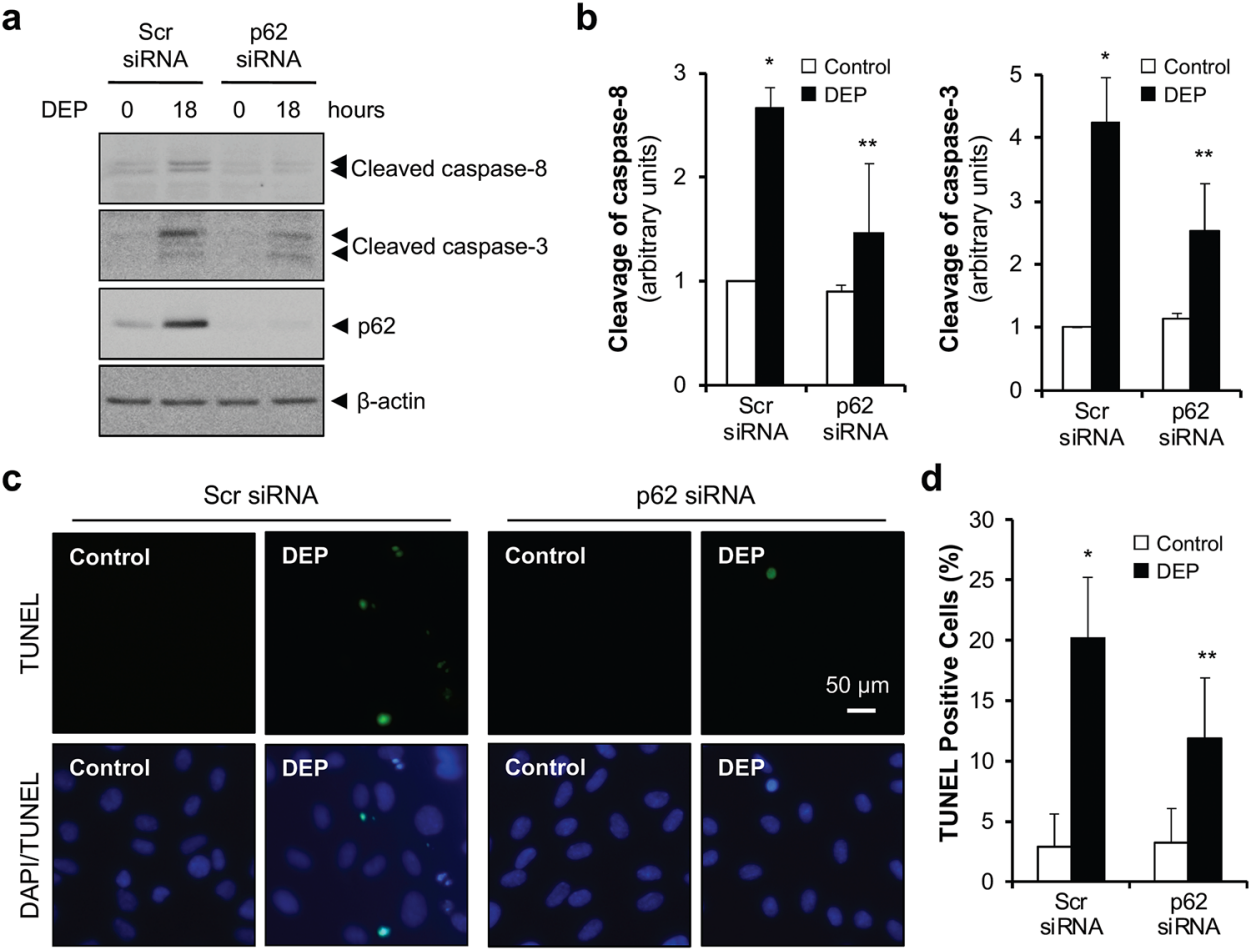
p62 depletion suppresses DEP-induced apoptosis. (**a**) HUVECs were transfected with scrambled or p62 siRNA and exposed to DEP (70 μg/ml) for 18 h. Cleavage of caspase-8 and caspase-3 were analyzed by immunoblotting. (**b**) Quantification of cleaved caspase-8 and cleaved caspase-3 normalized to β-actin. Results are presented as means ± SD (*n* = 3). Statistical analysis was performed using one-way ANOVA. *P < 0.02 versus scrambled siRNA with control. **P < 0.05 versus scrambled siRNA with DEP. (**c**) HUVECs were transfected with either scrambled or p62 siRNA and exposed to DEP (70 μg/ml). After 24 h, cells were subjected to TUNEL staining. (**d**) Quantification of TUNEL-positive cells. TUNEL-positive apoptotic cells were counted and expressed as a percentage of total nuclear counts. Results are presented as means ± SD from five randomly selected fields. Statistical analysis was performed one-way ANOVA. *P < 0.02 versus scrambled siRNA with control. **P < 0.02 versus scrambled siRNA with DEP.

p62 is a multidomain protein that regulates formation of autophagosomes by interacting with LC3 through the LC3-interacting region (LIR)^34^. As p62 interacts with LC3 and caspase-8 in autophagosomes of DEP-exposed HUVECs (Fig. 5a and b), we hypothesized that inhibition of p62 would impair caspase-8 activation. After depletion of p62 by siRNA transfection, caspase-8–caspase-3 cascade activation and HUVECs apoptosis were examined. DEP-induced activation of caspase-8 and caspase-3 was significantly reduced in p62 siRNA-transfected HUVECs compared with scrambled siRNA-transfected HUVECs (Fig. 6a and b). We observed the same p62 depletion effect on caspase-8 and caspase-3 activation in HAECs (see Supplementary Fig. S3 online). Furthermore, the proportion of TUNEL-positive cells induced by DEP exposure was dramatically decreased in p62 siRNA-transfected HUVECs compared with scrambled siRNA-transfected HUVECs (Fig. 6c and d). These data identify an important role of accumulated autophagosomes in DEP-induced caspase-8 − caspase-3 activation and subsequent apoptosis in HUVECs.

## Discussion

**Figure 7:**
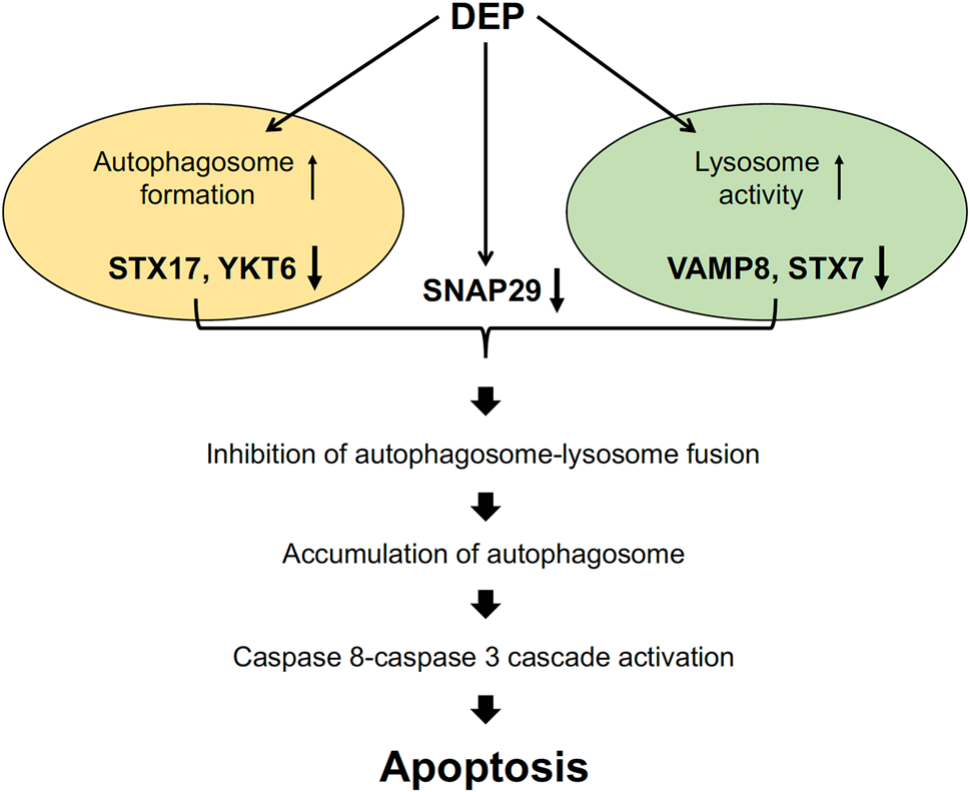
Schematic model for DEP-induced ECs apoptosis. In response to DEP exposure, HUVECs initiate the autophagic pathway, form the autophagosome, and increase lysosomal activity. However, DEP suppress expression of several SNARE proteins and block autophagosome maturation, leading to caspase-8 activation and subsequent caspase-3-dependent apoptosis.

The major findings of this study are that DEP initiate the autophagic process, inducing autophagosome formation but inhibiting autophagosome fusion with lysosomes, leading to accumulation of autophagosomes, which activates the caspase-8–caspase-3 cascade. These results, confirmed in two endothelial cell types (HUVECs and HAECs) reveal a novel mechanism of DEP-induced ECs apoptosis, which depends on autophagosome accumulation and caspase-8 activation. Based on our observations, we propose that autophagosomes and caspase-8 are important for ECs apoptosis and likely for blood vessel dysfunction. In this model, DEP initiate autophagy by increasing phosphorylation of beclin-1 at serine 93 and increasing protein levels of p62 and LC3II, resulting in autophagosomes formation. At the same time, DEP suppress expression of two SNARE complexes and inhibit the fusion of autophagosomes to lysosomes. Subsequently, the LC3–p62–caspase-8 complex forms in the accumulated autophagosomes, resulting in activation of the caspase-8–caspase-3 cascade and subsequent apoptosis (Fig. 7).

In this study, we used high concentration of DEP of which diameter is < 2.5 μm to study the mechanism of ECs apoptosis. It is reasonable to use the PM2.5 in ECs apoptosis because such a small PM penetrates the respiratory system and circulates through blood vessels causing vascular dysfunction^7^. However, it is not clear whether direct treatment of high concentration of DEP to HUVECs recapitulates the phenomena in which inhaled DEP exposure affects to blood vessel in vivo. Thus, though our study reveals a novel mechanism of DEP-induced ECs apoptosis, further study is necessary to examine whether these observations occur in vasculature in vivo system.

During the past several decades, multiple types of DEP have been used to study the adverse effects on various cell types^18,35–37^. We opted to generate DEP using a diesel engine, analyze their chemical composition, and continue to use these DEP in our future. Similarly, we generated gasoline exhaust particles (GEP) from a gasoline engine, analyzed their chemical composition, and performed RNA sequencing analysis in HUVECs exposed to GEP^21^. The total chemical component of DEP (94,760.88 μg/m^3^) (see Supplementary Table S1 online) is almost threefold higher than that of GEP (33,568.71 μg/m^3^)^21^. The percentages of elements (0.07%), ions (2.12%), and elemental carbon (0.52%) in DEP (see Supplementary Table S1 online) were similar to those of GEP (element: 0.10%, ion: 2.27%, elemental carbon: 1.30%)^21^. However, the percentage of organic carbon in DEP (92.05%) (see Supplementary Table S1 online) was higher than that of GEP (78.10%)^21^. In the present study, the effects of individual elements or ions on HUVECs is not clear. However, based on previous studies, cadmium (Cd) is a candidate chemical component as it is responsible for DEP-induced cardiovascular disease^38,39^. The specific contributions of different DEP components to ECs apoptosis should be examined in detail in future studies.

We observed that protein levels of p62 and LC3BII were increased by DEP treatment until 24 h after exposure (Fig. 2c and d). As inhibition of the autophagic process stabilizes p62 and LC3BII proteins, which are normally degraded by the autolysosome^40^, this finding, together with the RFP-LC3B-GFP and LC3 turnover assay (Fig. 4a–d), clearly indicates that DEP block the autophagic process at the autophagosome stage. The autophagosome functions as a platform for the iDISC, which induces non-canonical caspase-8 activation^16^. Consistent with this, we observed that DEP treatment caused caspase-8 activation, subsequent caspase-3 activation (Fig. 5c and d), and caspase-8–dependent apoptosis (Fig. 5c–g). These processes are highly dependent on expression of p62 (Fig. 6), which contains multiple functional motifs/domains, including the LC3-interacting region and the ubiquitinated protein-interacting UBA domain^34^. A previous study demonstrated that p62 induces HUVECs apoptosis by autophagy-independent pathways in some contexts^20^. Tumor necrosis factor α enhances binding of p62 to protein kinase C (PKC)ζ through interaction with the Phox/Bem1p (PB1) domain, which activates PKCζ leading to subsequent downstream activation of c-Jun N-terminal kinase and the subsequent caspase-8–caspase-3 cascade^20^. Thus, we examined the potential involvement of this pathway in HUVECs exposed to DEP. However, PKCζ was not activated in DEP-exposed HUVECs (see Supplementary Fig. S4 online), ruling out involvement of the PKCζ pathway in DEP-induced caspase-8 activation and apoptosis. To our knowledge, the present study is the first to define the role of caspase-8 in DEP induction of ECs apoptosis and delineate the mechanism by which initiation of autophagy induces apoptosis rather than survival in DEP-exposed HUVECs.

Autophagosome maturation, in which an autophagosome fuses with a lysosome and forms an autolysosome to degrade the cytosolic compartment, is necessary for cell survival under various stress conditions^2,23^. This fusion process is driven by various proteins, including SNARE complexes^2^. SNARE complexes are formed by autophagosome-localized STX17 and, SNAP29 and lysosome-localized VAMP8, or by autophagosome-localized YKT6 and, SNAP29 and lysosome-localized STX7^3,4^. The activities of these proteins are post-transcriptionally modified. For example, SNAP29, the most important SNARE for autophagosome maturation^3^, is O-GlcNAcylated by O-linked β-N-acetylglucosamine (O-GlcNAc) transferase (OGT), decreasing SNARE complex assembly^41^. STX17, the autophagosome-localized binding partner of STX29, can be modified via acetylation by the histone acetyltransferase CREBBP/CBP and the deacetylase HDAC2^5^. Under starvation conditions, deacetylated STX17 expedites assembly of the STX17–SNAP29–VAMP8 complex, promoting autophagosome maturation^5^. In contrast to these prior studies, our findings demonstrated that DEP exposure decreases SNARE complex protein levels (Fig. 4e and f). Recently, Liu et al. demonstrated that PM2.5 collected from Zhanjiang China impair autophagic flux by suppressing expression of lysosomal-associated membrane protein-2 (LAMP-2) and STX17^42^. Though the concept of this study appears to be similar to the present study, Liu et al. examined the expression of only two proteins, LAMP-2 and STX17, which regulate fusion between the autophagosome and lysosome^3,43^ and did not further investigate how accumulated autophagosomes led to apoptosis. Thus, the present study is a significant advance in understanding the molecular mechanisms of DEP-induced ECs death involving SNARE proteins and caspase-8.

Blockage of autophagosome maturation occurs in various human diseases^1,44^. In the past decade, many studies have evaluated the efficacy of the autophagy-inducing drugs, but most of these drugs target the initiation of autophagy or lysosome activation^45–47^. Because DEP initiate autophagy and activate lysosomes but suppress autophagosome maturation, protection of DEP-exposed blood vessels is likely to require another autophagy-inducing drug that targets autophagosome maturation. Currently, the mechanism for suppression of SNARE proteins expression in DEP-exposed HUVECs is unknown. However, proteasome is a potential target because previous studies showed that 20S proteasome degrades SNAP29 and STX17^48^ and inhibition of proteasome reverses the impairment of SNARE complex assembly by increasing SNAP25 and is beneficial for alleviating neurodegeneration^49^. The limitation of our study is that we did not delineate the mechanism of SNARE proteins suppression. Thus, it is worth determining the molecular mechanism of this observation and identifying the regulatory molecule as a therapeutic target for DEP-caused vascular dysfunction. Alternatively, adenovirus-mediated overexpression of some SNARE proteins is another potential target as the efficacy of using the LAMP-2B-expressing adenovirus has therapeutic effects in a murine model of Danon disease^50^.

In conclusion, the present study indicates that blockage of autophagosome maturation leads to p62-dependent caspase-8–caspase-3 cascade activation and subsequent apoptosis in DEP-exposed HUVECs. Because suppression of SNARE protein levels (STX17, VAMP8, SNAP29, YKT6, and STX7) inhibits autophagosome maturation, therapeutic strategies to maintain SNARE protein levels could protect blood vessels against DEP exposure.

## Methods

### DEP generation

Generation of fine particles from diesel engine exhaust was performed as previously described^21,51^. Briefly, engine exhaust particles were produced with a diesel engine (498 cc, DG8500SE, Hi-Earns Mechanical and Electrical Co., Ltd., Changzhou, China) and collected on filters using a PM2.5 low-volume sampler (URG-2000-30EH, URG, Chapel Hill, NC, USA) at a flow rate of 16.7 L/min for 30 min. The mass of PM2.5 was determined based on the weight of the filter, which was equilibrated at 21 ± 2 °C and relative humidity of 35 ± 5% for 24 h before and after collection. Mass concentration (μg/m^3^) was calculated by dividing the collected PM2.5 mass (μg) by the volume of collected air (m^3^). Analyses of ions, elements, and carbonaceous species were performed as described previously^21^. To study the cellular effects of DEP, DEP were collected on to a glass fiber filter (Pall Corporation, Port Washington, NY, USA) and extracted with dimethylsulfoxide (DMSO, Sigma-Aldrich, St. Louis, MO, USA). Extracted DEP were filtered through a PTFE syringe filter (Sartorius AG, Germany) before treatment of HUVECs.

### Cell culture and material sources

HUVECs and HAECs were purchased from Lonza (Walkersville, MD, USA) and cultured with an endothelial cell growth medium-2 (EGM-2) bullet kit (Lonza) without addition of vascular endothelial growth factor^21^. HUVECs were cultured in cell culture dishes precoated with 0.2% gelatin (Sigma-Aldrich) and used at passages 4 to 6. Antibodies against phospho-Beclin-1(Ser93), Beclin-1, p62, LC3B, Cathepsin D, STX17, cleaved caspase-8, and cleaved caspase-3 were purchased from Cell Signaling Technology (Beverly, MA, USA). Antibodies against VAMP8, SNAP29, and YKT6 were purchased from Santa Cruz Biotechnology Inc. (Santa Cruz, CA, USA). The STX7 antibody was purchased from Bethyl Laboratories Inc. (Montgomery, TX, USA). Antibodies against α-tubulin and β-actin were purchased from Sigma-Aldrich. Bafilomycin A1 was purchased from Sigma-Aldrich. Z-IETD-FMK were purchased from abcam (Cambridge, MA, USA).

### Determination of IC_50_ concentration

The IC_50_ DEP concentration was calculated according to dose-dependent cytotoxic effects of DEP^21^. HUVECs were seeded overnight in 96-well cell culture plates precoated with 0.2% gelatin at a density of 1 × 10^4^ cells/well. Subsequently, cells were exposed to various concentrations of DEP or the corresponding volume of DMSO for 24 h. After incubation with WST-1 reagent (Takara Bio Inc., Shiga, Japan) for an additional 4 h, absorbance at 420 nm was measured on a SpectraMax Plus 384 microplate reader (Molecular Devices, San Jose, CA, USA). After subtracting the absorbance of culture medium plus WST-1 reagent in the absence of cells, cell viability was calculated by dividing the absorbance of each concentration in the DEP-treated group with that of the corresponding volume of DMSO vehicle. Cell viability in the corresponding DMSO-treated group was considered to be 100%.

### Immunoblotting

Western blotting was performed as previously described^21^. Cells were lysed in cell lysis buffer (Cell Signaling Technology) supplemented with protease inhibitor cocktail (Sigma-Aldrich). Equal amounts of proteins were separated by sodium dodecyl sulfate–polyacrylamide gel electrophoresis and proteins were transferred onto polyvinylidene difluoride membranes (Millipore, Billerica, MA). After blocking in 5% nonfat milk (Santa Cruz Biotechnology Inc.) in 0.1% Tween 20-containing Tris-buffered saline (TBS) for 1 h, membranes were incubated overnight at 4 °C with appropriate primary antibodies. After washing three times with 0.1% Tween 20-containing TBS, membranes were incubated with horseradish peroxidase (HRP)-conjugated secondary antibody (GenDEPOT, Baker, TX) for 1 h. After washing with 0.1% Tween 20-containing TBS, signals were visualized with an ImageQuant LAS4000 mini system (GE Healthcare, Chicago, IL, USA) using either SuperSignal™ West Femto Maximum Sensitivity Substrate (Thermo Fisher Scientific, Waltham, MA, USA) or Western Blotting Luminol Reagent (Santa Biotechnology Inc.). Densitometric analysis was performed using Image J software (ver. 1.53). The representative image of western blotting was presented from at least three independent experiments. Uncropped blots are presented in Supplementary Fig. S5 online.

### Adenovirus autophagy assay

Adenoviruses expressing GFP-LC3B or RFP-LC3B-GFP were generated by Sirion Biotech GmbH (Martinsried, Germany). HUVECs were seeded onto 6-well plates precoated with 0.2% gelatin and infected with adenovirus expressing either GFP-LC3B or RFP-LC3B-GFP for 12 h. After removing the adenoviral particles, cells were exposed to DEP for 18 h, and the number of GFP-LC3 puncta per cell and the red/green signal were observed using fluorescence microscopy (EVOS M5000 Imaging System, Invitrogen, Carlsbad, CA, USA).

### Terminal deoxynucleotidyl transferase dUTP nick end labeling (TUNEL) assay

HUVECs were seeded onto 6-well plates precoated with 0.2% gelatin and transfected with the indicated siRNA. Cells were exposed to DEP for 24 h, washed with PBS, and fixed with 4% paraformaldehyde for 10 min at room temperature. To quantify apoptotic cells, the DeadEnd™ Fluorometric TUNEL system (Promega, Madison, WI) was used according to the manufacturer’s instructions. Cells were stained with DAPI prior to imaging with fluorescence microscopy (EVOS M5000 Imaging System, Invitrogen). The number of TUNEL-positive apoptotic cells was counted from five randomly selected fields. Subsequently, the percentage of TUNEL-positive apoptotic cells relative to total cells counted was calculated.

### Lysosome activity assay

Lysosome-dependent proteolytic activity was measured using DQ™ Red BSA (Molecular Probes, Eugene, OR, USA). The degradation of DQ™ Red BSA by hydrolases in active endo-lysosomes results in highly red fluorescent products that can be observed by confocal microscopy^29^. Cells were seeded onto Lab-TEKTM II Chamber Slides (Nalge Nunc International, Rochester, NY) precoated with 0.2% gelatin overnight. Cells then were exposed to DEP for 12 h. After washing with PBS, cells were further incubated with DQ™ Red BSA (10 μg/ml) for 4 h and fixed in 4% paraformaldehyde for 10 min. After washing with PBS, slides were mounted in ProLong^®^ Gold antifade reagent with DAPI (Invitrogen) and immediately analyzed under confocal microscopy (FVS3000-ORS, Olympus Corporation, Tokyo, Japan).

### siRNA transfection

HUVECs were transiently transfected with scrambled or p62 siRNA using OPTI-MEM (Invitrogen) and Lipofectamine RNAiMAX reagents (Invitrogen) according to the manufacturer’s instructions. Scrambled siRNA and human p62 siRNA were purchased from Santa Cruz Biotechnology Inc.

### Immunofluorescence staining

HUVECs were washed with cold PBS, fixed in 4% paraformaldehyde for 10 min, permeabilized with 0.1% Triton X-100 in PBS for 5 min, and blocked with 10% normal goat serum (Cell Signaling Technology) in PBS for 1 h. Cells were incubated with rabbit anti-LC3B antibody (1:100 dilution, Cell Signaling Technology), mouse caspase-8 antibody (1:50, dilution, Santa Cruz Biotechnology Inc.), and guinea pig anti-p62 antibody (1:100 dilution, Progen, Wayne, PA, USA) in 1% bovine serum albumin (BSA)-containing PBS overnight at 4 °C. Cells were then washed with 0.5% Tween-20-containing PBS and incubated with Alexa Fluor 488-conjugated goat anti-rabbit IgG (H&L) secondary antibody (Invitrogen), Alexa Fluor 568-conjugated goat anti-mouse IgG (H&L) secondary antibody (Invitrogen), and Alexa Fluor 405-conjugated goat anti-guinea pig IgG (H&L) secondary antibodies (abcam). After three washes with 0.5% Tween-20 containing PBS, cells were mounted using ProLong^®^ Gold antifade reagent (Invitrogen) and immediately analyzed under a confocal microscope (FVS3000-ORS, Olympus Corporation). The representative image was presented from three independent experiments.

### Statistics

Results are presented as means ± SD. Statistical significance between two groups was evaluated using a two-tailed Student’s t test. Statistical significance between more than two groups was evaluated using one-way ANOVA*. P* < 0.05 was considered significant.

## Supplementary Information

Supplementary Information.

## Data Availability

All data needed to evaluate the conclusions are present in the paper.
