# Supplementary material for: Diesel exhaust particles induce human umbilical vein endothelial cells apoptosis by accumulation of autophagosomes and caspase-8 activation
Source: Sci Rep. 2022 Oct 3;12:16492. doi: 10.1038/s41598-022-21044-3 (PMC9529885; doi:10.1038/s41598-022-21044-3)

Supplementary Information

Diesel exhaust particles induce human umbilical vein endothelial cells apoptosis by accumulation of autophagosomes and caspase-8 activation

Geun-Young Kim[1, *], Inkyo Jung[1], Minhan Park[2], Kihong Park[2],

Seung Hee Lee[1], Won-Ho Kim[1]

[1] Division of Cardiovascular Disease Research, Department of Chronic Disease Convergence Research, Korea National Institute of Health, Cheongju, Republic of Korea

[2] School of Earth Science and Environmental Engineering, Gwangju Institute of Science and Technology, Gwangju, Republic of Korea

[*]**Correspondence:** Geun-Young Kim, Ph.D.

Division of Cardiovascular Disease Research, Department of Chronic Disease Convergence Research, Korea National Institute of Health, 187 Osongsaengmyeng2-ro, Osong-eub, Heungdeok-gu, Cheongju-si, Chungcheongbuk-do 28159, Republic of Korea.

Tel.: + 82-43-719-8655; Fax: + 82-43-719-8689

E-mail: geunyoungkim77@gmail.com

Table S1. Chemical components

	Elements	Ions	Elemental Carbon	Organic Carbon	Others	Total
**Component (μg/m^3)**	68.36	2,004.56	495.02	87,227.23	4,965.71	94,760.88
**% of total mass**	0.07	2.12	0.52	92.05	5.24	100

Table S2. Elemental components

	Component ($\mu g/m^3$)	% of total mass
**Al**	9.96	14.57
**Fe**	3.78	5.53
**Mg**	2.32	3.39
**Zn**	6.49	9.49
**Sr**	0.03	0.04
**Na**	5.79	8.47
**P**	24.95	36.50
**Cr**	0.39	0.57
**Mn**	0.14	0.20
**Co**	0.13	0.19
**Ni**	0.32	0.47
**Cu**	0.76	1.11
**Se**	2.30	3.36
**Pb**	0.09	0.13
**Ca**	10.91	15.96

Table S3. Ionic components

	Component (μg/m^3)	% of total mass
**SO_4^{2-}**	297.87	14.86
**NO_3^-**	1,215.65	60.65
**Cl^-**	22.38	1.12
**Na^+**	318.61	15.89
**NH_4^+**	1.34	0.07
**K^+**	82.06	4.09
**Mg^{2+}**	18.90	0.94
**Ca^{2+}**	47.75	2.38

Fig. S1. LC3B turnover assay reveals that DEP impair autophagic flux. (a) HAECs were pre-incubated with Bafilomysin A1 (Baf A1, 100 nM) for 3 h and exposed to DEP (70 μg/ml) for an additional 12 h. LC3B level was measured by immunoblotting. (b) Quantification of LC3BII levels normalized to β-actin. Results are presented as means ± SD (n = 3). Statistical analysis was performed using one-way ANOVA. ***P* < 0.02 versus no treatment.

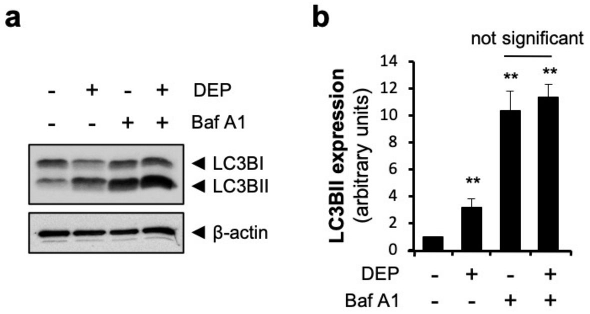

Fig. S2. DEP suppress the expression of SNARE proteins. (a) HAECs were exposed to DEP (70 μg/ml) for 24 h and protein levels of STX17, VAMP8, SNAP29, YKT6, STX7, and β-actin were measured by immunoblotting. (b) Quantification of STX17, VAMP8, SNAP29, YKT6, and STX7 levels normalized to β-actin. Results are presented as means ± SD (n = 3). Statistical analysis was performed using two-tailed Student's t test. **P* < 0.05 versus control. ***P* < 0.02 versus control.

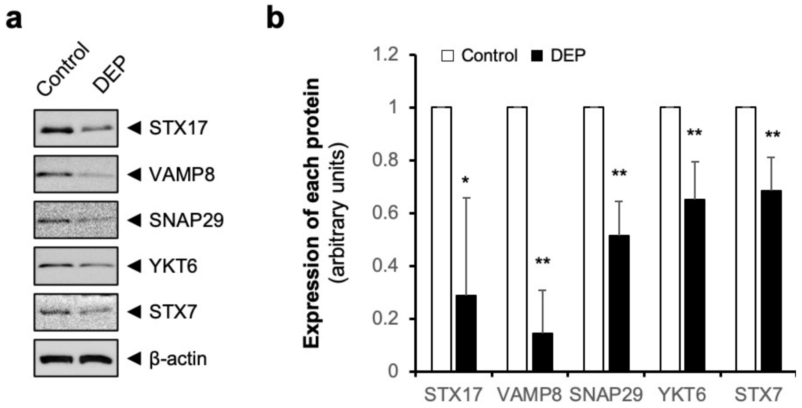

Fig. S3. p62 depletion suppresses DEP-induced caspase-8 and caspase-3 activation. (a) HAECs were transfected with scrambled or p62 siRNA and exposed to DEP (70 μg/ml) for 18 h. Cleavage of caspase-8 and caspase-3 were analyzed by immunoblotting. (b) Quantification of cleaved caspase-8 and cleaved caspase-3 normalized to β-actin. Results are presented as means ± SD (n = 3). Statistical analysis was performed using one-way ANOVA. *$P < 0.02$ versus scrambled siRNA with control. **$P < 0.05$ versus scrambled siRNA with DEP.

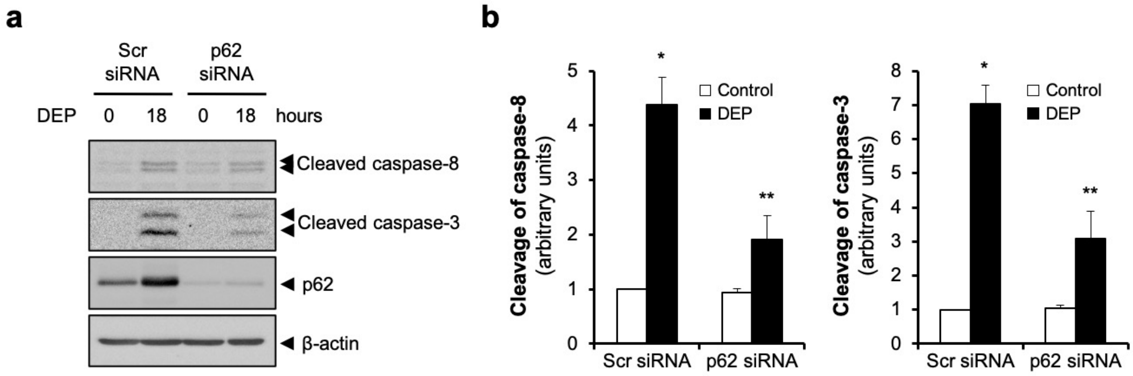

Fig. S4. DEP do not activate PKCζ. (a) HUVECs were exposed to DEP (70 µg/ml) for the indicated times and phosphorylation of PKCζ at Thr410/403 was analyzed by immunoblotting. Stimulation with TNFα (10 ng/ml, 30 min) was used as a positive control. Uncropped blots are presented in Supplementary Fig. S2 online. **(b)** Quantification of PKCζ phosphorylation normalized to total PKCζ. Results are presented as means ± SD (n = 3). Statistical analysis was performed using One-way ANOVA. **P* < 0.02 versus no treatment. Antibodies against phospho-PKCζ(Thr410/403) and PKCζ were purchased from Cell Signaling Technology (Beverly, MA, USA) and Santa Cruz Biotechnology Inc. (Santa Cruz, CA, USA), respectively. TNFα was purchased from Roche (Basel, Switzerland).

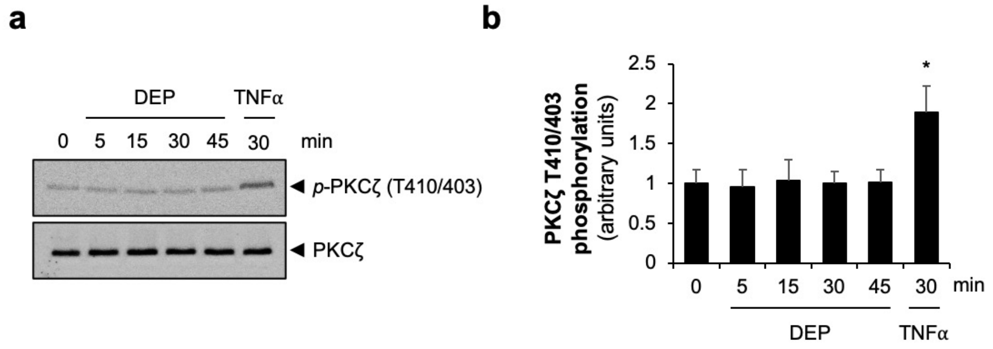

Fig. S5. Original blots in western blotting. The membranes were cut prior to hybridization with antibodies; thus, the images of full-length blots cannot be provided. Area of the blots which was used in Figures were highlighted by dotted-red delineation.

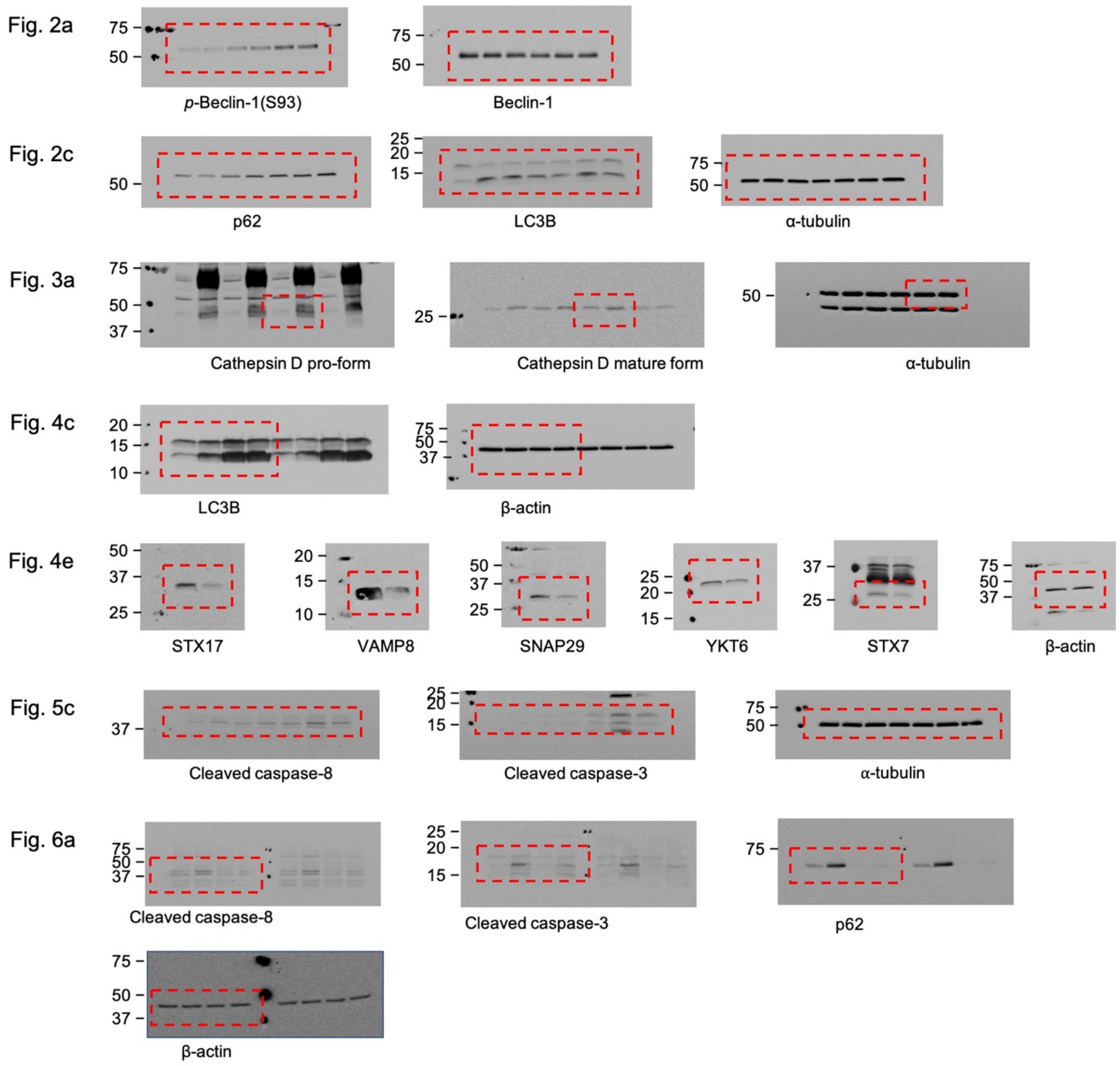

Fig. S1a

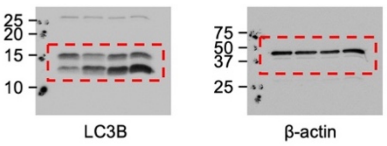

Fig. S2a

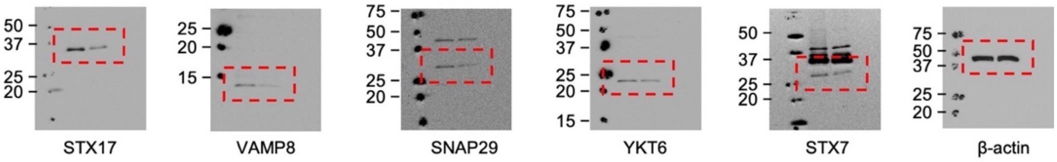

Fig. S3a

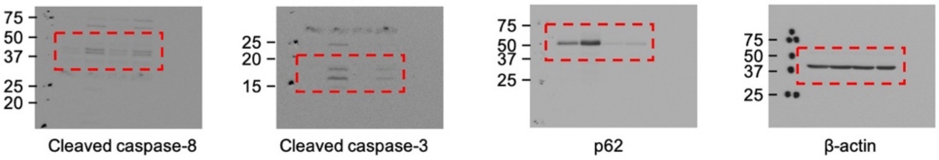

Fig. S4a

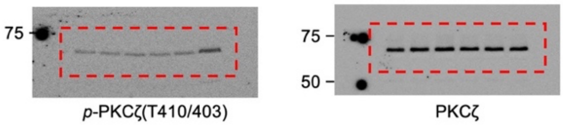

Supplement: Supplementary file 1 — Supplementary Information. [file 41598_2022_21044_MOESM1_ESM.pdf]
